# Limitations of the Farquhar–von Caemmerer–Berry Model in Estimating the Maximum Electron Transport Rate: Evidence from Four C_3_ Species

**DOI:** 10.3390/biology14060630

**Published:** 2025-05-29

**Authors:** Zipiao Ye, Wenhai Hu, Shuangxi Zhou, Piotr Robakowski, Huajing Kang, Ting An, Fubiao Wang, Yi’an Xiao, Xiaolong Yang

**Affiliations:** 1 Institute of Biophysics, Math & Physics College, Jinggangshan University, Ji’an 343009, China; yezp@jgsu.edu.cn (Z.Y.); wangfubiao@jgsu.edu.cn (F.W.); 2 School of Life Science, Jinggangshan University, Ji’an 343009, China; huwenhai@jgsu.edu.cn; 3 Department of Biological Sciences, Macquarie University, Sydney, NSW 2000, Australia; shuangxi.zhou@dpird.wa.gov.au; 4 Faculty of Forestry and Wood Technology, Poznan University of Life Sciences, Wojska Polskiego 71E, 60-625 Poznan, Poland; piotr.robakowski@up.poznan.pl; 5 Key Laboratory of Crop Breeding in South Zhejiang, Wenzhou Academy of Agricultural Sciences, Wenzhou 325006, China; kanghuajing@126.com; 6 College of Bioscience and Engineering, Jiangxi Agriculture University, Nanchang 330045, China; anting_6918@163.com; 7 School of Life Sciences, Nantong University, Nantong 226019, China; 8 State Key Laboratory of Environmental Chemistry and Ecotoxicology, Research Center for Eco-Environmental Sciences, Chinese Academy of Sciences, Beijing 100085, China

**Keywords:** Farquhar–von Caemmerer–Berry (FvCB) model, maximum electron transport rate for CO_2_ assimilation (*J*_A-max_), CO_2_ response, observation-modelling intercomparison

## Abstract

The study evaluates the accuracy of two FvCB model sub-models (I and II) in estimating the maximum electron transport rate for CO_2_ assimilation (*J*_A-max_) by comparing estimated values with observed maximum electron transport rates (*J*_f-max_) in four C_3_ species: *Triticum aestivum* L., *Silphium perfoliatum* L., *Lolium perenne* L., and *Trifolium pratense* L. Significant discrepancies were found between *J*_A-max_ estimates from sub-model I and observed *J*_f-max_ values for *T. aestivum*, *S. perfoliatum*, and *T. pratense* (*p* < 0.05), with sub-model I overestimating *J*_A-max_ for *T. aestivum*. Sub-model II consistently produced higher *J*_A-max_ estimates than sub-model I. This study highlights limitations in the FvCB sub-models, particularly their tendency to overestimate *J*_A-max_ when accounting for electron consumption by photorespiration (*J*_O_), nitrate reduction (*J*_Nit_), and the Mehler reaction (*J*_MAP_). An alternative empirical model provided more accurate *J*_f-max_ estimates, suggesting the need for improved approaches to model photosynthetic electron transport. These findings have important implications for crop yield prediction, ecological modeling, and climate change adaptation strategies, emphasizing the need for more accurate estimation methods in plant physiology research.

## Simple Summary

Accurately measuring how plants convert sunlight into energy through photosynthesis is crucial for predicting crop yields and understanding plant responses to climate change. Scientists often use mathematical models, like the FvCB model, to estimate the maximum rate of electron transport in plants—a key factor in photosynthesis. However, this study found that two widely used versions of the FvCB model often overestimate this rate when tested on four common plant species, including wheat and ryegrass. By comparing model predictions with direct measurements, this research revealed that the models fail to account for energy losses caused by processes like photorespiration and nutrient absorption. The empirical model proposed by Ye et al. provided more accurate and reliable estimates. These findings highlight the need to improve existing models to better predict plant growth under changing environmental conditions. This work will help farmers, ecologists, and policymakers make more informed decisions about crop management and climate adaptation strategies, ensuring food security and ecosystem resilience in a warming world.

## 1. Introduction

As global environmental changes intensify, the ability to accurately estimate plant photosynthetic rates has become increasingly critical for predicting and adapting to future climate conditions. The light-driven electron transport chain in the photosynthetic apparatus is the fundamental force powering plant growth and development. A key parameter is the maximum electron transport rate for CO_2_ assimilation (*J*_A-max_), which represents the peak rate under light-saturated conditions and reflects the upper limit of energy conversion during photosynthesis. Therefore, accurate determination of *J*_A-max_ is essential for understanding plant responses to environmental factors such as light, CO_2_ concentration, temperature, nutrition, and water availability [1,2,3,4,5,6].

To estimate *J*_A-max_, both indirect and direct methods have been developed. The indirect method, based on the Farquhar–von Caemmerer–Berry (FvCB) model, estimates *J*_A-max_ by fitting the CO_2_ response curve of photosynthesis (*A*_n_–*C*_i_ curve) under light-saturated conditions. The FvCB model, which considers the enzymatic kinetics of Rubisco and the chemometrics of RuBP regeneration in C_3_ species, is widely used for this purpose [2,3,5,6,7,8,9,10,11]. Direct methods include the non-rectangular hyperbolic model (NRH model) and a mechanistic model of the light response of electron transport rate (*J–I* curve) developed by Ye et al. [12,13]. These models estimate or calculate *J*_max_ by fitting the *J–I* curve, and the latter model considers light energy absorption and pigment molecule excitation in photosynthesis. However, these models are typically used to estimate *J*_max_ under constant CO_2_ conditions and do not investigate the CO_2_ response of photosynthesis in plants.

With rising atmospheric CO_2_ levels, understanding C_3_ plant responses to elevated CO_2_ is crucial. The FvCB model is a key tool for investigating this response and has been extensively applied to study plant physiology under various conditions [4,5,6,14,15,16,17,18,19,20,21,22]. The FvCB model estimates key parameters like *J*_A-max_, the maximum carboxylation rate (*V*_cmax_), triose phosphates utilization (*V*_TPU_), day respiration rate (*R*_d_), and mesophyll conductance (*g*_m_), which are vital for crop yield prediction, ecological modeling, and global carbon cycling [4,5,6,17,20,23,24,25,26]. However, *J*_A-max_ in the FvCB model is indirectly estimated, necessitating a modeling–observation intercomparison approach to assess its accuracy under different conditions.

The FvCB model’s development involves two equations for estimating *J*_A-max_ by fitting the *A*_n_–*C*_i_ curve of C_3_ plants. One assumes RuBP regeneration is limited by NADPH alone (sub-model I) [2,5,6,7,27,28,29], while the other assumes co-limitation by NADPH and ATP (sub-model II) [5,8,23,27,30,31]. Sub-model I is often favored for its accuracy in estimating *J*_A-max_ across diverse environmental conditions. In contrast, sub-model II is employed by others to explore the photosynthetic physiology and ecology of C_3_ plants under varied environmental conditions [5,8,23,27,30,32].

However, in practice, it is uncertain whether RuBP regeneration in C_3_ plants is limited by NADPH alone or co-limited by NADPH and ATP, leading to uncertainty in model selection and *J*_A-max_ estimation accuracy. Therefore, it is essential to verify the consistency of *J*_A-max_ values estimated by the two sub-models with observed values, especially under varying environmental conditions.

Our objective in this study is to simultaneously measure the *A*_n_–*C*_i_ and *J*–*C*_i_ curves for *Triticum aestivum* L., *Silphium perfoliatum* L., *Lolium perenne* L., and *Trifolium pratense* L. at saturating irradiance (*I*_sat_), fit the *A*_n_–*C*_i_ curves using both FvCB sub-models to estimate *J*_A-max_ values, and compare these with observed values (*J*_f-max_) to determine the more accurate sub-model. It also seeks to investigate reasons and solutions if estimated *J*_A-max_ values differ from observed values, providing new criteria for evaluating RuBP regeneration limitation assumptions in the FvCB model.

## 2. Material and Methods

### 2.1. Plant Material

Four species were used as test materials. *T. aestivum* (Jimai 22) is a widely cultivated wheat variety in Shandong Province, China. *S. perfoliatum*, native to the tallgrass prairie regions of North America, was introduced to China from Korea in 1979 by the Institute of Botany, Chinese Academy of Sciences, and is now used as a high-quality forage species. *L. perenne* (cv. Zhongxin 830) was developed through multigenerational hybridization between hexaploid and octoploid parental lines (H4372 × woH90) by the Crop Breeding and Cultivation Research Institute of the Chinese Academy of Agricultural Sciences. *T. pratense* is native to China and is widely used for forage and ornamental purposes. The seeds used in this study were obtained from Jinan Tianshundifeng. Agricultural Technology Co., Ltd., Jinan, China. The experimental site was located at Yucheng Station in the southwest of Yucheng County, Chinese Academy of Sciences, Shandong Province. The plants were sown on 15 October 2022 and maintained under standard field conditions. Data collection was performed on sunny days from 28 April 2023 to 10 May 2023. *T. aestivum* was in the booting to flowering stage, with an approximate plant height of 60–70 cm, and the flag leaf was selected for testing. *S. perfoliatum* was also in vigorous vegetative growth, with an approximate plant height of 30 cm, and the second leaf fully unfolded from top was selected for testing. *L. perenne* was in the booting stage, with an approximate plant height of 1.3 m. The first leaf beneath the flag leaf was selected for testing. *T. pratense* was in a period of vigorous vegetative growth with an approximate plant height of 15 cm, and mature leaves at the top were selected for testing.

### 2.2. Gas Exchange and Chl Fluorescence Measurement

From 28 April 2023 to 10 May 2023, *A*_n_–*I* curves were measured for each study plant with an open-path gas exchange system (LI-6400; Li-Cor, Lincoln, NE, USA), equipped with a leaf chamber fluorometer (6400-40; Li-Cor). Measurements were taken between 9:30 and 11:30 and between 14:30 and 17:00 on sunny days. The ambient [CO_2_] concentration was maintained at 420 μmol mol^−1^ for 15 or 13 light levels in the following order (first to last): 2000, 1800, 1600, 1400, 1200, 1000, 800, 600, 400, 200, 150, 100, 80, 50, and 0 μmol m^−2^ s^−1^ for *L. perenne* and *T. aestivum*; 1600, 1400, 1200, 1000, 800, 600, 400, 200, 150, 100, 80, 50, and 0 μmol m^−2^ s^−1^ for *T. pratense*; and 1400, 1200, 1000, 800, 600, 400, 200, 150, 100, 80, 50, and 0 μmol m^−2^ s^−1^ for *S. perfoliatum*. The plants were allowed to acclimate to changes in light intensity for approximately 2–3 min before measurements were logged; it took about 50 min to complete an entire *A*_n_–*I* curve. After data collection, we employed a mechanistic model of *A*_n_–*I* in PMSS (http://photosynthetic.sinaapp.com, accessed on 15 May 2024) to simulate the *A*_n_–*I* curves, determining the saturating irradiance (*I*_sat_) to be 1200, 900, 2000, and 2000 μmol m^−2^ s^−1^ for *T. aestivum*, *S. perfoliatum*, *L. perenne*, and *T. pratense*, respectively. Then *A*_n_–*C*_i_ and *J*–*C*_i_ curves were simultaneously recorded at saturating irradiance for 12 CO_2_ concentrations in the following order (first to last measurement): 1600, 1400, 1200, 1000, 800, 600, 420, 300, 200, 100, 60, and 0 μmol mol^−1^. To ensure steady-state conditions, the plants were given approximately 5 min to acclimate to ambient CO_2_ (420 μmol mol^−1^) in the gas exchange chamber before beginning each *A*_n_–*C*_i_ and *J*–*C*_i_ curve, and then logged. It took approximately 45 min to complete a single *A*_n_–*C*_i_ and *J*–*C*_i_ curve. The two FvCB sub-models in PMSS (http://photosynthetic.sinaapp.com, accessed on 16 May 2024) were then used to simulate the *A*_n_–*C*_i_ curves and obtain the *J*_A-max_ values for the four C_3_ plants.

### 2.3. J_A-max_ Estimated by the FvCB Model

Electron transport and concomitant proton transfer in the chloroplast’s thylakoids produces NADPH and ATP [10]. For steady-state C_3_ leaf photosynthesis, the carbon assimilation rate is assumed to be limited either by Rubisco-catalyzed carboxylation or by regeneration of RuBP, which is controlled by the electron transport rate [2,5,6,7,10,28,29,31,33,34]. When calculating the electron transport-limited rate of CO_2_ assimilation (*A*_j_), there are many equations for estimating *J*_A-max_ [5,10]. Among these, two FvCB sub-models are commonly used to estimate the *J*_A-max_ value [5,10,31].

Sub-model I can be expressed as follows [2,5,6,7,10,28,29,31]:

(1) $$A_{j}=J\frac{C_{i}-\Gamma_{*}\;}{4C_{i}+8\Gamma_{*}}-R_{d}$$

where *C*_i_ is the intercellular CO_2_ concentration, *Γ*_∗_ is the CO_2_ compensation point in the absence of day respiratory rate, and *R*_d_ is the daily respiration rate.

In sub-model II, the limitation is co-determined by both NADPH and ATP availability, and the equation takes a slightly different form [5,10,23,27,30,31,35]:

(2) $$A_{j}=J\frac{C_{i}-\Gamma_{*}\;}{4.5C_{i}+10.5\Gamma_{*}}-R_{d}$$

At the saturation irradiance, the value of *J* estimated by sub-model I and sub-model II represents the maximum electron transport rate for carbon assimilation (*J*_A-max_). In addition, through simple mathematical analysis, both sub-models I and II exhibit asymptotic functions without extreme values. They, while similar, provide different estimates for *J*_A-max_ based on assumptions regarding the biochemical limitations of photosynthesis. Mathematical analysis shows that the *J*_A-max_ estimated by sub-model I is greater than that estimated by sub-model II, because the denominator in Equation (2) is larger than that in Equation (1) for any same value of *Γ*_∗_ and *C*_i_. The difference in the denominator reflects the additional ATP requirement per unit of CO_2_ fixation when accounting for photorespiration and other processes.

### 2.4. An Empirical Model for the CO_2_ Response of Electron Transport Rate (Model 1)

The empirical model of CO_2_ response of the electron transport rate for photosynthetic organisms (including C_3_, C_4_, CAM species, algae, and photosynthetic bacteria) [36] is:

(3) $$J_{f}=\alpha \frac{1-\beta C_{i}}{1+\gamma C_{i}}+J_{0}$$

where *J* is electron transport rate, *α, β,* and *γ* are three coefficients independent of *C*_i_, and *J*_0_ is the electron transport rate, while *C*_i_ = 0 μmol·mol^−1^.

Saturation CO_2_ concentration (*C*_isat_) corresponds to the maximum electron transport rate (*J*_f-max_), and *J*_f-max_ can be obtained as follows:

(4) $$C_{\mathrm{isat}}=\frac{\sqrt{\frac{\left(\beta +\gamma \right)}{\beta }}-1}{\gamma }$$

and

(5) $$J_{f-\max}=\alpha {\left(\frac{\sqrt{\beta +\gamma }-\sqrt{\beta }}{\gamma }\right)}^{2}+J_{0}$$

By fitting the *J*–*C*_i_ curves using Equation (3), the values of *C*_isat_ and *J*_f-max_ can be obtained from Equations (4) and (5), respectively.

These values can then be directly compared with the observed values to verify the accuracy of Equation (3). No significant differences were found between the estimated *J*_f-max_ values and observed *J*_f-max_ values (see Table 1). Consequently, our results indicate that Equation (3) is an effective model for simulating *J*–*C*_i_ curves and for calculating *C*_isat_ and *J*_f-max_.

### 2.5. Statistical Analyses

The two FvCB sub-models and the empirical *J*–*C*_i_ model proposed by Ye et al. (available via the PMSS web platform, http://photosynthetic.sinaapp.com) were used to simulate the *A*_n_–*C*_i_ and *J*–*C*_i_ curves to determine the *J*_A-max_ values for the four plants. PMSS integrates these models and uses Simulated Annealing and the Metropolis Algorithm to fit photosynthetic parameters to user-provided data. Data are expressed as the means ± standard error. Student’s *t*-tests were used to compare the *J*_A-max_ values estimated by the two FvCB sub-models with the corresponding observed *J*_f-max_ values. Data were analyzed by one-way analysis of variance (ANOVA) at *p* < 0.05 (*p*-significance level) using SPSS 18.5 statistical software (SPSS, Chicago, IL, USA). Figures were generated using Origin 7.0 (Origin Lab, Northampton, MA, USA) and Adobe Illustrator CS6 (Adobe, San Jose, CA, USA). The goodness of fit between the experimental observations and the line of best fit obtained from mathematical modeling were assessed using the coefficient of determination (*R*^2^), calculated as *R*^2^ = 1 − SSE/SST, where SST is the total sum of squares, and SSE is the error sum of squares.

## 3. Results

### 3.1. A_n_–C_i_ Curve Analysis

**Figure 1:**
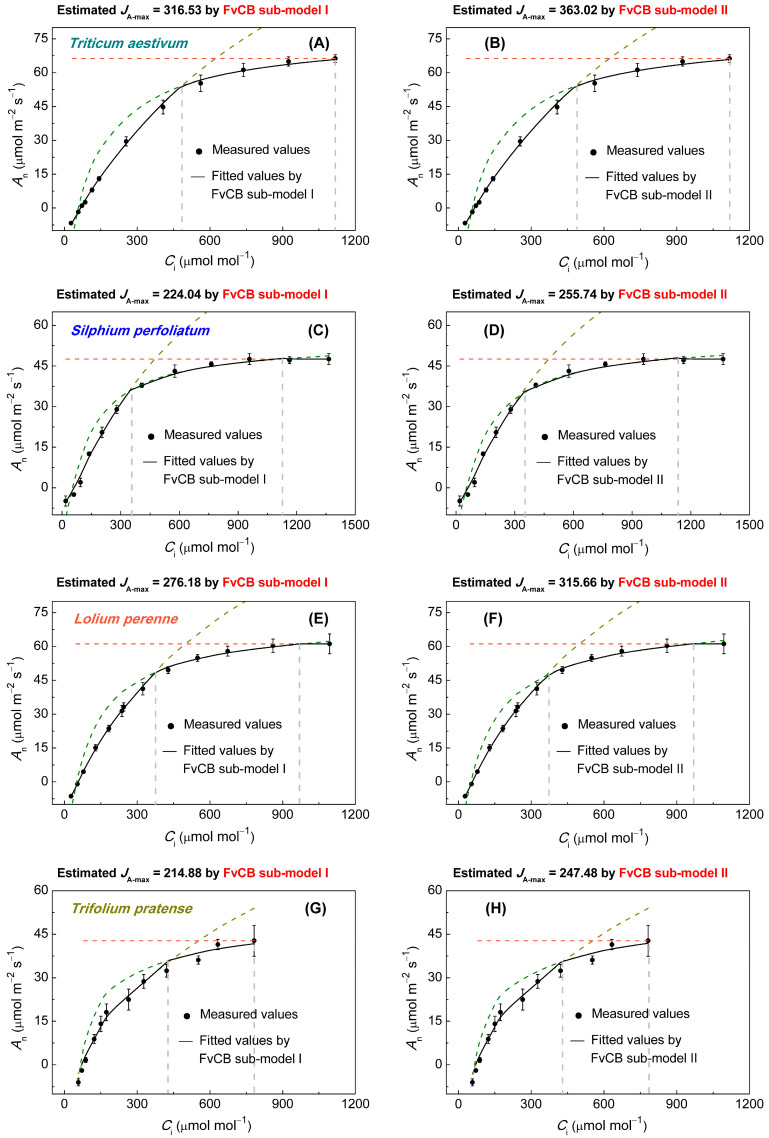
*A*_n_–*C*_i_ curves illustrating the CO_2_ response of photosynthesis for the four C_3_ species at 12 CO_2_ concentrations under saturating irradiance, namely (**A**,**B**) *Triticum aestivum*, (**C**,**D**) *Silphium perfoliatum*, (**E**,**F**) *Lolium perenne,* and (**G**,**H**) *Trifolium pratense*. Solid black dots represent observed experimental data. Data represent mean ± *SE*, *n* = 3. Solid lines represent lines of best fit modeled by the FvCB sub-model I (**A**,**C**,**E**,**G**) and sub-model II (**B**,**D**,**F**,**H**). *A*_n_–*C*_i_ curves are typically divided into three stages, including Rubisco-limited, RuBP-limited, and triose-phosphate-utilization-limited (*TPU*-limited).

The CO_2_ assimilation rates of all four C_3_ species showed a typical *A*_n_–*C*_i_ response under saturating irradiance (Figure 1). At CO_2_ concentrations below approximately 500 μmol·mol^−1^, *A*_n_ increased rapidly, reflecting the Rubisco-limited phase. As CO_2_ concentration continued to increase, *A*_n_ also increased, but at a slower pace, particularly for *T. aestivum* (Figure 1A,B), *S. perfoliatum* (Figure 1C,D), and *L. perenne* (Figure 1E,F), which reached a plateau, indicating *C*_i_ transit points from RuBP-limited to triose-phosphate-utilization-limited (*TPU*-limited) photosynthesis (*C*_i,TPU_). Notably, the carbon assimilation rate of *S. perfoliatum* showed a slight decrease after reaching *C*_i,TPU_ (Figure 1C,D).

### 3.2. Comparison of J_max_ Estimates

**Figure 2:**
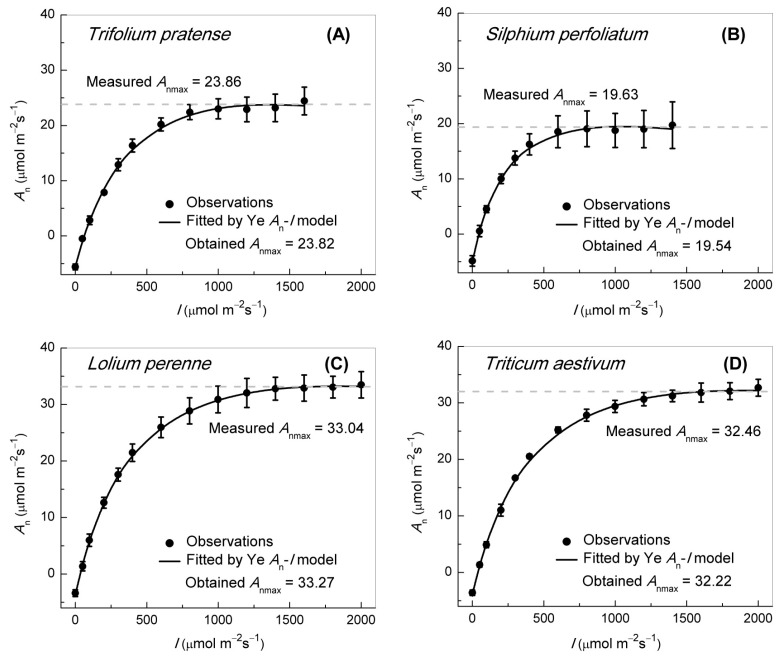
Light response (*A_n_*–*I*) curves of photosynthesis for the four C_3_ species at 13–15 light levels maintained at an ambient CO_2_ concentration of 420 μmol mol^−1^, namely (**A**) *Triticum aestivum*, (**B**) *Silphium perfoliatum*, (**C**) *Lolium perenne,* and (**D**) *Trifolium pratense*. Solid black dots represent observed experimental data. Data represent mean ± *SE*, *n* = 3–6. *A_n_*–*I* curves (**A**–**D**) are modeled by the Ye *A*_n_–*I* model.

**Figure 3:**
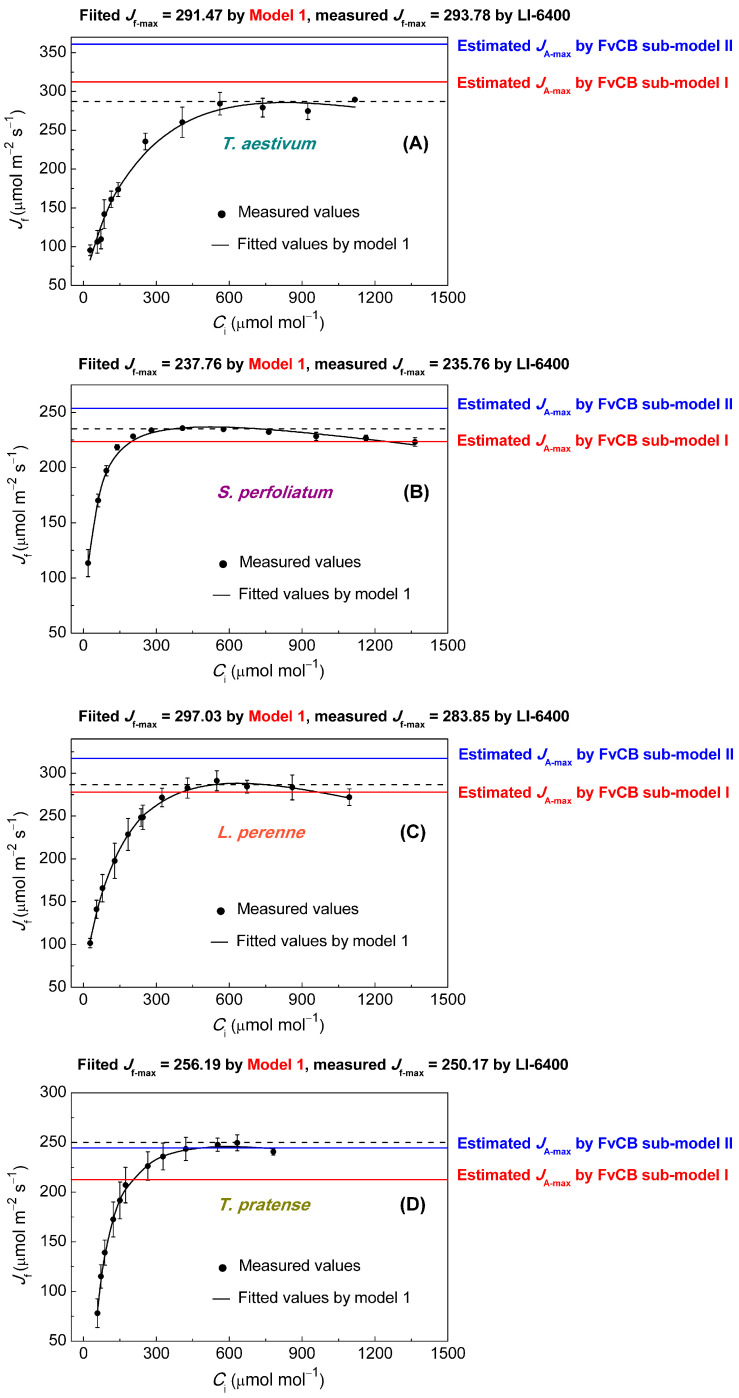
*J*–*C*_i_ curves showing the CO_2_ response of electron transfer rate for the four C_3_ species at 12 CO_2_ concentrations under saturating irradiance, namely (**A**) *Triticum aestivum*, (**B**) *Silphium perfoliatum*, (**C**) *Lolium perenne,* and (**D**) *Trifolium pratense*. Solid black dots represent observed experimental data. Data represent mean ± *SE*, *n* = 3. Solid black lines represent lines of best fit modeled by the empirical model proposed by Ye et al. [36]. The black dashed lines, blue solid lines, and red solid lines represent the measured values of *J*_f-max_ using LI-6400 and the estimated values of *J*_A-max_ using FvCB sub-model I and FvCB sub-model II, respectively.

Comparison of *J*–*C*_i_ curves under saturating irradiance (derived from fitted *A*_n_–*C*_i_ curves in Figure 2) revealed significant differences between observed *J*_max_ (*J*_f-max_) values and estimated *J*_max_ values using the two FvCB sub-models in all species (Figure 3). For *T. pratense*, sub-model I significantly underestimated *J*_max_ compared to observed values (*p* < 0.05) (Figure 3A, Table 1), while sub-model II did not (*p* > 0.05). For *S. perfoliatum*, both sub-models significantly differed from observed values (*p* < 0.05), with sub-model I underestimating and sub-model II overestimating *J*_max_ (Figure 3B, Table 1). For *L. perenne*, the *J*_max_ values estimated by sub-model I were not significantly different from observed values (*p* > 0.05), but sub-model II significantly underestimated *J*_max_ (*p* < 0.05) (Figure 3C, Table 1). For *T. aestivum*, both sub-models significantly overestimated *J*_max_ (*p* < 0.05) (Figure 3D, Table 1). Sub-model II consistently produced higher *J*_max_ estimates than sub-model I across all species. The *R*_d_ and *Γ*_∗_ parameters were identical between the two sub-FvCB models (Supplementary Table S1). In contrast, the Ye empirical *J*–*C*_i_ model accurately reproduced both the CO_2_ response trends of *J* and the *J*_max_ values for all four plants, showing no significant difference from measured values (*p* > 0.05) (Figure 3A–D, Table 1).

## 4. Discussion

### 4.1. Why Can Current Technology Validate the Estimation of J_A-max_ by the FvCB Model?

At present, advanced experimental methodologies, such as chlorophyll fluorescence-based techniques, provide a more comprehensive understanding of electron partitioning. As elucidated by von Caemmerer [10] and Long and Bernacchi [23], *J*_f_ supports not only *J*_A_, but also photorespiration (*J*_O_), nitrate-to-ammonium conversion (*J*_Nit_), and Mehler ascorbate peroxidase (MAP)-reaction-driven oxygen uptake (*J*_MAP_). This relationship can be expressed as *J*_f_ = *J*_A_ + *J*_O_ + *J*_Nit_ + *J*_MAP_.

This relationship underscores that *J*_f_ must exceed *J*_A_ under any environmental conditions. When *J*_A-max_ is inferred from the *A*_n_–*C*_i_ curve—a method that indirectly estimates *J*_A-max_—the sub-models consider only the electron transport rate dedicated to carbon assimilation (*J*_A-max_). This approach neglects other electron-consuming processes, including *J*_O_, *J*_Nit_, and *J*_MAP_. Conversely, the maximum *J*_f_ (*J*_f_-max) values, which are directly obtained from the *J*–*C*_i_ curve, represent the total electron transport rate (*J*_f_) originating from photosystem II (PSII). This differentiation is critical, because *J*_A_ represents just a portion of the overall electron flow, suggesting that the *J*_A-max_ derived from the FvCB sub-models should invariably be lower than the observed *J*_f-max_ across various environmental conditions. Hence, the *J*_A-max_ estimated by the two FvCB sub-models should consistently be lower than the observed *J*_f-max_. This criterion is fundamental for evaluating the credibility of the FvCB model’s estimation of *J*_A-max_.

### 4.2. What Explains the Discrepancies Between Estimated J_A-max_ and Observed J_f-max_?

The discrepancies between estimated *J*_A-max_ and observed *J*_f-max_ values, particularly for *T. aestivum*, highlight a need for a deeper examination of the underlying assumptions in the FvCB sub-models. For *T. aestivum*, the observed *J*_f-max_ values consistently fall below the *J*_A-max_ estimates from both sub-models (Figure 1A,B and Figure 3A; Table 1), posing difficulties in interpretation within the context of the FvCB model. When considering consumption of *J*_O_, *J*_Nit_, and *J*_MAP_, the *J*_A-max_ value derived from the FvCB model should be considerably lower than the observed *J*_f-max_ value, not alarmingly higher. From this, we can deduce that the *J*_A-max_ for *T. aestivum*, as estimated by the FvCB model, is unreasonable. Upon scrutinizing these results through the perspective of mathematical interpolation, it becomes evident that the coefficients for *C*_i_ and *Γ*_∗_ in Equation (1) might be overstated. It is only when these coefficients for *C*_i_ and *Γ*_∗_ are reduced that the estimated *J*_A-max_ has the potential to fall below the *J*_f-max_. For *S. perfoliatum* and *L. perenne*, analogous findings were observed; the *J*_A-max_ values were found to be overestimated when accounting for consumption of *J*_O_, *J*_Nit_, and *J*_MAP_ (Figure 1C–F and Figure 3B,C; Table 1).

For example, in the case of *T. aestivum*, when consumption of *J*_O_, *J*_Nit_, and *J*_MAP_ is neglected, the coefficient of *Γ*_∗_ in the denominator of Equation (1) must be adjusted to approximately 7.02 (a value less than 8) to ensure that the *J*_A-max_ value remains lower than the *J*_f-max_ values. This adjustment implies that 0.57 mol of CO_2_ is released in the photorespiratory pathway for every mol of RuBP oxygenated, which exceeds the theoretical value of 0.5 mol [7]. However, this conclusion conflicts with the widely accepted consensus that 1 mol of O_2_-bound RuBP releases 0.5 mol of CO_2_ [2,5,7,10,27,28,29,31,37]. Therefore, the claim that the amount of CO_2_ released in the photorespiratory pathway exceeds the theoretical value of 0.5 mol per mol of RuBP oxygenation appears to be unsupported.

To further evaluate this discrepancy, von Caemmerer’s theory provides an alternative perspective [10]. Specifically, if the Q cycle operates (H^+^/e^−^ = 3), the *J*_A-max_ for carbon assimilation can be estimated by $A_{j}=J\frac{C_{i}-\Gamma_{*}\;}{3C_{i}+7\Gamma_{*}}-R_{d}$. Under this assumption, the calculated *J*_A-max_ value is (240.02 ± 4.09) μmol m^−2^ s^−1^, which is significantly lower than the measured value of (293.78 ± 3.13) μmol m^−2^ s^−1^ (Table 1).

### 4.3. Can a More Comprehensive Framework Improve J_A-max_ or J_f-max_ Estimation?

Given the challenges associated with the FvCB sub-models, alternative approaches, such as the empirical model proposed by Ye et al. [36], offer a promising solution. This model directly calculates *J*_f-max_ from *J*–*C*_i_ curves, bypassing the need for indirect estimation via *A*_n_–*C*_i_ fitting. In this study, the empirical model produced *J*_f-max_ values that closely matched observed *J*_f-max_ values across all four species (Figure 3, Table 1). Most importantly, this approach accounts for the whole-chain electron transport rate, providing a more accurate representation of photosynthetic electron transport. This is especially relevant when considering other key parameters in the FvCB model, such as *V*_cmax_, *V*_TPU_, *R*_d_, and *g*_m_.

However, while the empirical model demonstrates superior accuracy, its lack of mechanistic detail limits its applicability in broader physiological studies. For instance, the biological significance of its coefficients in the empirical model remains unclear, and it does not explicitly link electron transport with ATP synthesis or the co-limitation of NADPH and ATP. As such, it should be regarded as a transitional tool, complementing the FvCB model while paving the way for more integrative approaches.

### 4.4. Should We Rethink the Estimation of J_A-max_?

The discrepancies in *J*_A-max_ estimations by the FvCB sub-models reveal the necessity for prudent application and interpretation of these models. When considering consumption of *J*_O_, *J*_Nit_, and *J*_MAP_, the consistent overestimation by sub-model II across four species and by sub-model I for *S. perfoliatum*, *L. perenne*, and *T. aestivum,* specifically for *T. aestivum* (Figure 3, Table 1) underscore the imperative to incorporate a deeper understanding of electron partitioning into model development. From a mathematical perspective, it is expected that the *J*_A-max_ values derived from fitting the *A*_n_–*C*_i_ curves of the four plant species using $A_{j}=J\frac{C_{i}-\Gamma_{*}\;}{3C_{i}+7\Gamma_{*}}-R_{d}$ would be lower than the corresponding *J*_f-max_ values. Similarly, the *J*_A-max_ values obtained by fitting the *A*_n_–*C*_i_ curves of *S. perfoliatum* and *L. perenne* using this equation are also anticipated to be lower than their respective *J*_f-max_ values.

### 4.5. How Does Overestimating J_A-__max_ Affect Agricultural and Environmental Research?

The implications of overestimating *J*_A-max_ extend across multiple scientific and practical domains, with significant consequences for both agricultural and environmental research. In agricultural contexts, inflated *J*_A-max_ values can lead to unrealistic yield projections, potentially resulting in suboptimal resource allocation and misguided management practices. Farmers and policymakers relying on these overestimations may make decisions that do not align with actual crop performance, leading to inefficiencies in resource use and potential economic losses.

In ecological modeling, inaccuracies in *J*_A-max_ estimates can significantly compromise our understanding of carbon flux dynamics and energy conversion efficiencies. This distortion can affect predictions of carbon sequestration capacity and plant responses to environmental changes, ultimately undermining the accuracy of climate models and ecological forecasts. Such inaccuracies may also hinder efforts to predict and mitigate the impacts of climate change on natural ecosystems.

Furthermore, the potential consequences for climate change adaptation strategies are particularly concerning. Inaccurate *J*_A-max_ assessments could lead to flawed predictions of plant resilience and adaptability under elevated CO_2_ concentrations and changing environmental conditions. This, in turn, could hinder the development of effective strategies for maintaining ecosystem stability and ensuring food security in the face of global climate change. Misguided adaptation strategies based on overestimated *J*_A-max_ values may fail to address the actual challenges posed by climate change, potentially exacerbating vulnerabilities in agricultural and natural systems.

While our study highlights these limitations in the examined species, the broader applicability of these findings requires further verification through additional research encompassing a wider range of plant species and environmental conditions. Future studies should focus on developing more accurate estimation methods and refining existing models to better capture the complex physiological processes underlying photosynthetic efficiency. By improving the precision of *J*_A-max_ estimates, researchers can enhance the reliability of predictions in agricultural and environmental research, ultimately supporting more informed decision-making and sustainable resource management.

## 5. Conclusions

This study evaluated the accuracy of two widely used sub-models of the FvCB model in estimating the maximum electron transport rate for CO_2_ assimilation across four C_3_ plant species. Our results revealed that both sub-models frequently overestimated the electron transport rate, particularly in *T. aestivum*, highlighting key limitations in their current formulations. By comparing these estimates with observed values obtained from direct measurements, we demonstrated that the empirical model proposed by Ye et al. provided more accurate and reliable estimates. These findings underscore the importance of refining photosynthetic models to more accurately reflect the physiological realities of plant electron transport. Improved models are essential for advancing research in plant physiology, enhancing crop yield predictions, and developing effective responses to climate change.

## Figures and Tables

**Table 1:** Comparison of estimated *J*_A-max_ values from fitting *A*_n_–*C*_i_ curves with the FvCB sub-models, *J*–*C*_i_ curves using the empirical model proposed by Ye et al. [36], and observed *J*_f-max_ values from LI-6400 for four C_3_ species (mean ± *SE*, *n* = 3). Estimated *J*_A-max_ and observed *J*_f-max_ values within one plant that are significantly different (*p* < 0.05) are marked with different superscript letters (e.g., 214.88 ± 3.31 ^b^ and 247.48 ± 3.60 ^a^), while those that are not significantly different (*p* > 0.05) share the same superscript letter (e.g., 247.48 ± 3.60 ^a^ and 250.17 ± 4.33 ^a^). Units of *J*_A-max_ and *J*_f-max_: μmol m^−2^ s^−1^.

Species	Fitted *J*_A-max_ Values by FvCB Sub-Model I	Fitted *J*_A-max_ Values by FvCB Sub-Model II	Fitted *J*_f-max_ Values by Empirical Model	Observed *J*_f-max_ Values by Li-6400
*Triticum aestivum*	316.53 ± 5.42 ^b^	363.02 ± 6.07 ^a^	291.47 ± 0.65 ^c^	293.78 ± 3.13 ^c^
*Silphium perfoliatum*	224.04 ± 2.47 ^c^	255.74 ± 2.73 ^a^	237.76 ± 1.36 ^b^	235.76 ± 0.98 ^b^
*Lolium perenne*	276.18 ± 7.20 ^b^	315.66 ± 8.57 ^a^	297.03 ± 10.23 ^b^	283.85 ± 3.36 ^b^
*Trifolium pratense*	214.88 ± 3.31 ^b^	247.48 ± 3.60 ^a^	256.19 ± 6.17 ^a^	250.17 ± 4.33 ^a^

## Data Availability

*R*_d_ and *Γ*_∗_, parameters derived from the FvCB two sub-models, are available in Supplementary Table S1.

## Supplementary Materials

The following supporting information can be downloaded at https://www.mdpi.com/article/10.3390/biology14060630/s1, Table S1: FvCB model-derived parameters *R*_d_ and *Γ*_∗_.
