# Supplementary material for: Limitations of the Farquhar–von Caemmerer–Berry Model in Estimating the Maximum Electron Transport Rate: Evidence from Four C3 Species
Source: Biology (Basel). 2025 May 29;14(6):630. doi: 10.3390/biology14060630 (PMC12189644; doi:10.3390/biology14060630)
Supplement: Supplementary file 1 [file biology-14-00630-s001.zip › biology-3634026-supplementary.pdf]

Supplementary Materials

Limitations of the FvCB Model in Estimating the Maximum Electron Transport Rate: Evidence from Four C_3 Species

Zipiao Ye [1,†,‡], Wenhai Hu [2,‡], Shuangxi Zhou [3], Piotr Robakowski [4], Huajing Kang [5], Ting An [6], Fubiao Wang [1], Yi'an Xiao [2,*] and Xiaolong Yang [7,8,*]

[1] Institute of Biophysics, Math & Physics College, Jinggangshan University, Ji'an 343009, China; yezp@jgsu.edu.cn (Z.Y.); wangfubiao@jgsu.edu.cn (F.W.)
[2] School of Life Science, Jinggangshan University, Ji'an 343009, China; huwenhai@jgsu.edu.cn
[3] Department of Biological Sciences, Macquarie University, Sydney, NSW 2000, Australia; shuangxi.zhou@dpird.wa.gov.au
[4] Faculty of Forestry and Wood Technology, Poznan University of Life Sciences, Wojska Polskiego 71E,
60-625 Poznan, Poland; piotr.robakowski@up.poznan.pl
[5] Key Laboratory of Crop Breeding in South Zhejiang, Wenzhou Academy of Agricultural Sciences, Wenzhou 325006, China; kanghuajing@126.com
[6] College of Bioscience and Engineering, Jiangxi Agriculture University, Nanchang 330045, China; anting_6918@163.com
[7] School of Life Sciences, Nantong University, Nantong 226019, China
[8] State Key Laboratory of Environmental Chemistry and Ecotoxicology,
Research Center for Eco-Environmental Sciences, Chinese Academy of Sciences, Beijing 100085, China
* Correspondence: iyanxiao@163.com (Y.X.); yangxl@ntu.edu.cn or xlyang@rcees.ac.cn (X.Y.)
† Current address: New Quality Productivity Research Center, Guangdong ATV College of Performing Arts,
Deqing 526631, China
‡ These authors contributed equally to this work.

Table S1 FvCB model-derived parameters R_d and Γ^*

Fitted parameters	*Triticum aestivum*	*Silphium perfoliatum*	*Lolium perenne*	*Trifolium pratense*
FvCB sub-model I				
R_d (μmol m^{-2} s^{-1})	2.98 ± 0.05	2.22 ± 0.04	2.59 ± 0.16	2.05 ± 0.22
Γ^* (μmol mol^{-1})	51.29 ± 1.18	45.46 ± 1.28	41.77 ± 2.69	49.40 ± 1.82
FvCB sub-model II				
R_d (μmol m^{-2} s^{-1})	2.98 ± 0.05	2.22 ± 0.04	2.59 ± 0.16	2.05 ± 0.22
Γ^* (μmol mol^{-1})	51.29 ± 1.18	45.46 ± 1.28	41.77 ± 2.69	49.40 ± 1.82
